# Case Report: Uterine mesothelial cyst in a nulliparous woman—diagnostic and clinical challenges of a rare benign lesion

**DOI:** 10.3389/fmed.2025.1672439

**Published:** 2025-09-18

**Authors:** Ying Shao, Yi Liu, Chun Yang

**Affiliations:** Department of Obstetrics and Gynecology, Union Hospital, Tongji Medical College, Huazhong University of Science and Technology, Wuhan, China

**Keywords:** uterine mesothelial cyst, preoperative diagnosis, etiology, clinical treatment, histopathology

## Abstract

Uterine mesothelial cysts (UMCs) are rare, benign lesions that arise from the mesothelial lining of the uterus and are only sporadically documented in the literature. Because of their extremely low incidence, the clinical presentation and imaging features of UMCs are often nonspecific, which makes preoperative diagnosis particularly challenging. We report the case of a 46-year-old nulliparous woman who presented with an intramural cystic mass of the uterus. Preoperative imaging suggested a benign lesion, but the differential diagnosis included cystic degeneration of leiomyoma, adenomyotic cyst, and endometriosis. The patient underwent a laparoscopic cystectomy, during which the cyst was completely excised without intraoperative complications. She recovered uneventfully and was discharged on postoperative day three. Histopathological examination confirmed the diagnosis of a uterine mesothelial cyst. At 1 month of follow-up, no recurrence was observed. This case highlights the importance of surgical excision for both definitive diagnosis and treatment, emphasizes the challenges in preoperative assessment, and contributes valuable clinical evidence to the limited body of knowledge on UMCs.

## Introduction

Mesothelial cysts are rare mesenchymal lesions, most of which are benign (1), with fewer than 5,000 documented cases worldwide since their first description by Mennemeyer and Smith in 1979 (2, 3). However, only six cases of mesothelial cysts in the uterine corpus have been documented. Although the exact pathogenesis of UMC remains unclear, it is generally suspected to involve a developmental disorder of mesothelial cells (4). Chronic inflammation of the peritoneum is widely considered the primary reason behind the development of mesothelial cysts, as it may promote cellular proliferation and the migration of mesothelial cells within peritoneal tissues (3). The presence of mesothelial cysts can be associated with conditions such as pelvic inflammatory disease, previous abdominal surgeries, and endometriosis (3, 5, 6). Karpathiou et al. (7) suggested that mesothelial cystic lesions are predominantly benign, although malignant transformation could potentially be related to chronic inflammation resulting from repeated interventions. Due to the unpredictable biological behavior and atypical clinical symptoms of UMC, early identification and aggressive treatments are essential.

## Case presentation

The patient was a 46-year-old married nulliparous Chinese woman who worked in an office environment. She denied smoking, alcohol consumption, or use of recreational drugs. She reported mild psychological distress due to a prolonged history of infertility but had no psychiatric disorders. Her family history was unremarkable for gynecological malignancies, endometriosis, or other cystic lesions. She sought medical attention at Wuhan Union Hospital on December 16, 2024, due to a persistent uterine mass. She had previously undergone curettage following a pregnancy and had no other notable medical conditions or prior surgeries. Although she occasionally experienced abdominal distension, she did not report abdominal pain, abnormal uterine bleeding, or dysmenorrhea.

### Physical examination

Upon bimanual pelvic examination, it was observed that the uterus was enlarged with thickened adnexa. The abdomen was found to be soft and non-sensitive, with the presence of an approximately 8 × 8 cm palpable mass.

### Laboratory and imaging results

**Figure 1:**
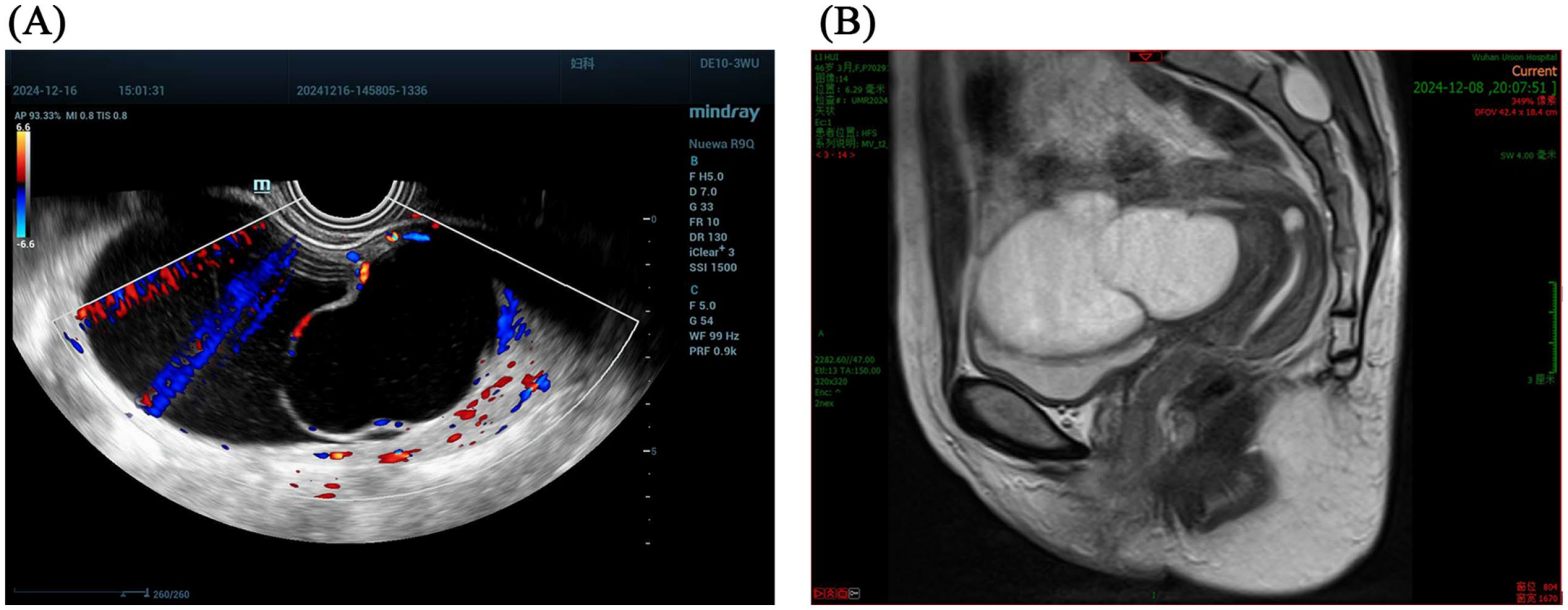
**(A)** Transvagina1 color Doppler ultrasound identified a hypoechoic mass characterized by well-defined margins and internal septations, with Doppler imaging showing blood flow. **(B)** Pelvic MRI confirmed the presence of a cystic structure in the anterior part of the uterine corpus.

Serum tumor markers, including CA125, CA19-9, CEA, and AFP, showed no specific abnormalities. Transvaginal color Doppler ultrasound identified a hypoechoic mass measuring approximately 90 × 75 mm in the anterior uterine corpus, characterized by well-defined margins and internal septations, with Doppler imaging showing blood flow within the mass (Figure 1A). Pelvic MRI confirmed the presence of a cystic structure in the anterior part of the uterine corpus, showing hyperintensity on T2 imaging and mild enhancement along its periphery (Figure 1B).

### Preoperative differential diagnosis

Based on the patient’s clinical presentation and preoperative imaging, we considered several entities that can manifest as an intramural cystic lesion of the uterus. The working differential included cystic degeneration of a leiomyoma, adenomyotic cyst, intramyometrial endometriosis, adenomatoid tumor, and other rare cystic lesions (e.g., Müllerian remnant or lymphangioma).

### Surgical treatment

**Figure 2:**
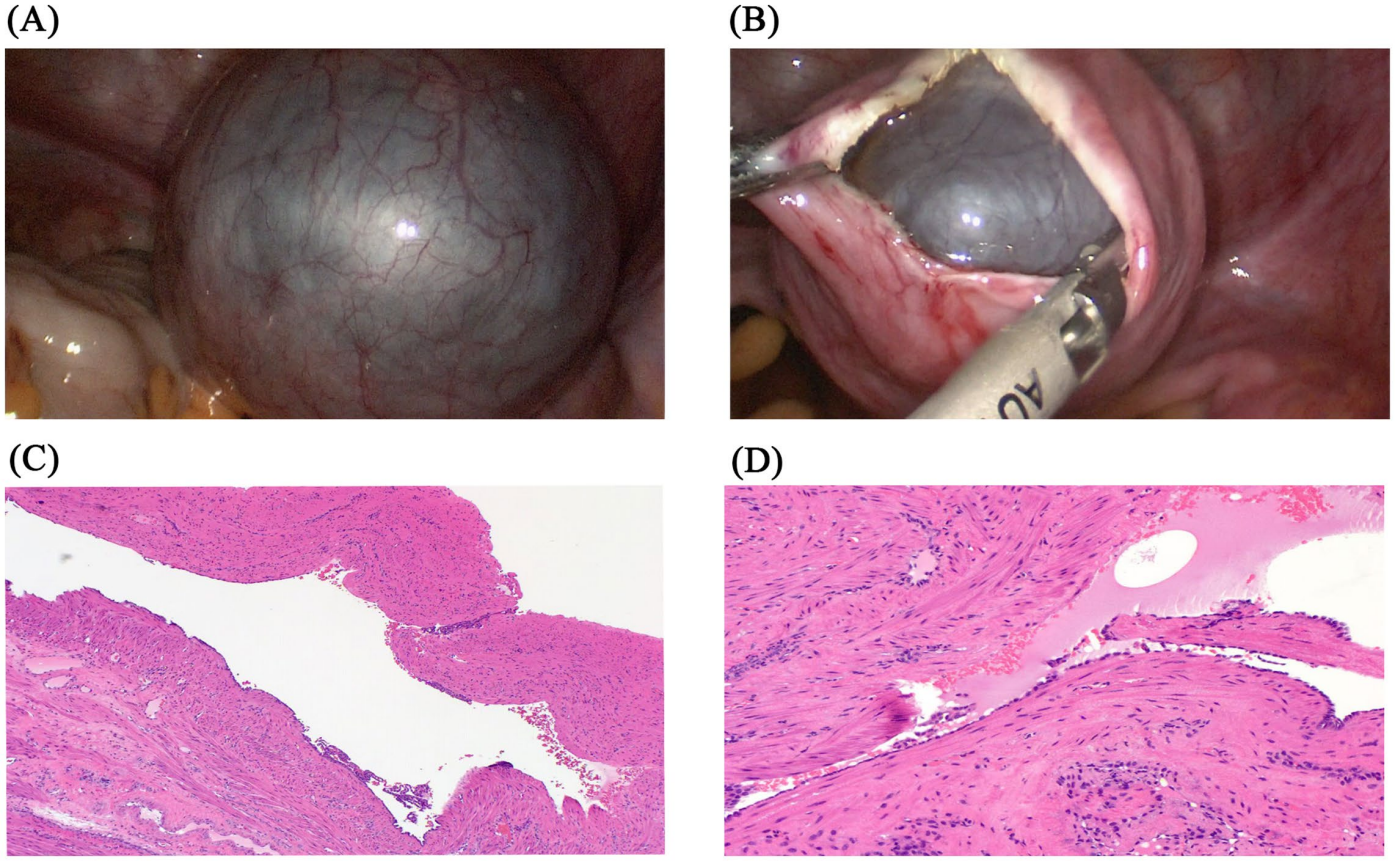
Examples of uterine mesothelial cysts pathology sections. **(A)** Uterine mesothelial cyst in the anterior uterine corpus. **(B)** Following incision of the myometrium, the intrauterine cyst is exposed. **(C)** Representative image of hematoxylin and eosin staining (40×) shows a single-layer lining of flattened epithelial cells. **(D)** Representative image of hematoxylin and eosin staining (100×) shows a transition from cuboidal to columnar morphology, with an absence of cilia.

During the surgery, a multilocular cyst filled with clear yellow fluid was discovered in the anterior wall of the uterus, with no other abnormalities detected (Figure 2A). The cyst was found near the endometrium and myometrium, exhibiting a smooth wall without nodules. Extensive adhesions to neighboring tissues made it challenging to separate the cyst from the uterus, requiring precise dissection along an unclear myometrial boundary (Figure 2B). The cyst was excised laparoscopically with no complications, and the patient was discharged within 3 days. Conclusive histological examinations ruled out cancer and confirmed the benign nature of the uterine cyst. Under the microscope, the cyst exhibited a single-layer lining of flattened epithelial cells, transitioning from cuboidal to columnar shapes, lacking cilia (Figures 2C,D). Additionally, no signs of cellular atypia, mitotic activity, or tissue necrosis were observed. The definitive pathology identified the lesion as a uterine mesothelial cyst.

### Rationale for surgical intervention

Although conservative strategies such as monitoring or cyst aspiration could have been considered, surgical excision was deemed more appropriate for several reasons. First, the imaging characteristics were non-specific, and histopathological confirmation was required to establish a definitive diagnosis. Second, aspiration or observation alone would carry a risk of recurrence and would not reliably exclude malignancy. Finally, laparoscopic excision provided both diagnostic and therapeutic benefits with low morbidity, making it the most reasonable management strategy.

### Follow-up and outcome

The patient successfully recovered from the surgery, and a follow-up examination after 1 month showed no signs of recurrence. The treatment timeline of the patient is presented in Table 1.

**Table 1:** Timeline.

Date	Clinical event	Notes
December 16, 2024	Presented to Wuhan Union Hospital with a uterine cystic lesion	Initial diagnosis undetermined
December 24, 2024	Transvaginal ultrasound and MRI performed	Revealed an intramural cystic mass, likely benign
December 25, 2024	Laparoscopic uterine cystectomy performed	Intraoperatively, a cyst within the myometrium was identified and completely excised
December 31, 2024	Histopathological examination confirmed a uterine mesothelial cyst	Rare benign lesion
February 1, 2025	One-month postoperative follow-up	Patient recovered well, no signs of recurrence

## Discussion

The clinical manifestations and imaging findings of uterine mesothelial cysts (UMCs) are often non-specific, leading to a relatively high rate of missed diagnoses. Uterine mesothelial cysts can develop on any peritoneal surface, including the round ligament, adnexa, mesentery, or peritoneum (2). In the uterus, the majority of reported cases involve the round ligament, often presenting a resemblance to an inguinal hernia. Instances of mesothelial cysts within the uterine corpus are exceptionally rare, and most mesothelial cysts appear benign (1). We found six cases of uterine mesothelial cysts after a Pubmed search of English-language studies published (Table 2). Six patients’ clinical presentations, locations and characteristics of the cysts, interventions, and outcomes were reviewed. Most cysts were detected during further evaluation for abdominal pain, and more than half were located in the posterior uterine wall. All patients were older than 39 years, except for one 27-year-old nulliparous woman reported by Ren et al. (4). With the exception of this patient, who underwent cyst excision alone to preserve fertility, nearly all others underwent total hysterectomy and achieved satisfactory outcomes, with no recurrence observed during follow-up. However, Mo et al. (6) also described a patient who experienced recurrence 3 months after cystectomy, which contrasts with the report by Ren.

**Table 2:** Case reports of uterine mesothelial cysts in the literature.

Author, year of publication, references	Age	Clinical presentation	Location of cyst	Mass size	Treatment	Follow-up
Momeni et al., 2014 (14)	47	Abdominal distention, abdominal tenderness, and constipation	Left cornual region of the uterine fundus	A 26–28 cm multiloculated cyst	Total abdominal hysterectomy, and bilateral salpingo-ophorectomy	NED at 1 year
Mishra et al., 2016 (2)	40	Abdominal pain	Uterine margin	Few small cysts	Laparoscopic-assisted vaginal hysterectomy	NM
Mo et al., 2019 (6)	44	None	The surface of posterior uterine wall	A 10 cm cystic mass	Laparoscopic uterine cystectomy	Recurrence at 3 months
	The patient was re-admitted 2 years later	Subjectively palpable abdominal mass	Right myometrium of the uterus	A 9 cm thin-walled cyst	Total hysterectomy and bilateral salpingectomy	NED at 3 months
Ren et al., 2023 (4)	27	Subjectively palpable abdominal mass	Posterior uterine wall	A 9 cm multiloculated cyst	Laparoscopic uterine cystectomy	NED at 2 years
Arslan et al., 2024 (16)	41	Abdominal pain	Posterior uterine wall	A 6 cm cystic lesion	Total laparoscopic hysterectomy and bilateral salpingectomy	NM
Lin et al., 2024 (5)	39	Dysmenorrhea	The surface of the posterior uterine wall	A 4 cm multiloculated cyst	Laparoscopic uterine cystectomy	NED at 1 month

NM, not mentioned; NED, no evidence of disease.

Many patients diagnosed with UMC have a prior history of pelvic inflammatory disease, endometriosis, or abdominal surgeries (3, 5, 6). Several studies suggest that uterine mesothelial cysts may have a congenital origin, originating from invagination and metaplasia of mesothelial cells or residual endodermal tissues within the uterus (8–11). Factors such as dust, chronic irritation or inflammation (2, 12, 13), endometriosis (14), and previous abdominal surgeries (14) are known to induce hyperplastic and neoplastic changes in mesothelial cells, potentially enhancing their proliferation and migration in underlying peritoneal tissues (14, 15).

Uterine mesothelial cysts generally present without specific clinical manifestations. Patients may be asymptomatic or exhibit symptoms such as abdominal masses (4), pelvic pain, abdominal distention, menorrhagia (6) or constipation (5, 16). The rarity of these cysts and the nonspecific nature of their symptoms pose challenges in preoperative identification.

Imaging plays a central role in the diagnostic process of uterine mesenchymal neoplasms (UMC). Various imaging techniques are indispensable for a precise diagnosis of UMC. In asymptomatic cases, serial ultrasound imaging is crucial for observing fluctuations in cyst dimensions and monitoring benign cystic mesothelioma (6). Computed tomography (CT) and magnetic resonance imaging (MRI) are highly effective in identifying and delineating uterine lesions (17). However, pathological analysis stands out as the gold standard for definitive confirmation of UMC (5, 16).

Microscopic evaluation typically reveals cysts surrounded by smooth muscle bundles, lined with a cuboidal-to-flat epithelial layer lacking cilia (16). Immunohistochemistry lacks a universally accepted standard; therefore, combining multiple markers enhances accuracy (4), calretinin is widely utilized due to its specificity and sensitivity to mesothelial cells (18). Additionally, HBME-1 (4), WT1, and cytokeratin 5/6 (6) are valuable for identifying mesothelial differentiation (19, 20). WT1 is shown to be less sensitive compared to calretinin (18). In various studies, the lining cells exhibited focal positive for WT1 (2, 16), while PAX8 expression remains inconsistent, yielding no definitive conclusions (2, 5). Test for estrogen receptor (ER), progesterone receptor (PR), and GATA3 typically yield negative results (4, 5, 16).

Given the extremely low incidence and limited number of published cases, no standardized treatment approach for uterine mesothelial cysts has been established. Treatment options encompass hormonal therapies, percutaneous aspiration or sclerotherapy guided by ultrasound, laser ablation, and chemotherapy, and various surgical interventions (21). However, despite these strategies, the recurrence rates are notably high (14, 21, 22). Ultrasound-guided aspiration typically offers only transient relief as a rapid reaccumulation occurs (23). En bloc surgical excision, though ideal, presents technical challenges due to the thin cyst walls and multicystic structures, increasing the risk of recurrence and potential damage to healthy uterine muscle (4, 5). Long-term monitoring is advisable due to the likelihood of recurrence and the potential for misdiagnosis (4, 5). For older patients or those not desiring pregnancy, hysterectomy—either total or partial—is recommended as the definitive treatment (4, 5).

In view of the patient’s long-standing history of infertility in this case, the potential impact of uterine mesothelial cysts on reproductive outcomes is of clinical interest. Although the exact relationship between UMCs and infertility remains unclear, an intramural cystic lesion may distort the uterine cavity, compromise endometrial receptivity, or interfere with implantation. Surgical excision in this case was performed not only for diagnostic and therapeutic purposes but also to restore normal uterine anatomy and preserve reproductive potential. In this case, the duration of follow-up was limited to 1 month, longer follow-up is needed to assess fertility outcomes, and further reports are required to clarify the association between UMCs and infertility. In conclusion, this study emphasizes the clinical manifestations, diagnosis, and therapeutic options for UMC, providing crucial insights for prompt detection and efficient management of this uncommon condition.

## Patient perspective

“I felt anxious when I realized that a cyst was found in my uterus, as I was worried about its impact on my fertility and health. Although I was initially hesitant about undergoing surgery, I decided to follow the doctor’s advice. My recovery was smooth, I am relieved with the outcome and hope my experience can encourage other women facing similar conditions.”

## Data Availability

The raw data supporting the conclusions of this article will be made available by the authors, without undue reservation.
